# The impact of innovative behaviors on academic misconduct among graduate students: a mediated moderation model

**DOI:** 10.3389/fpsyg.2023.1276700

**Published:** 2023-10-11

**Authors:** Peng Su, Mu He

**Affiliations:** ^1^School of Marxism, University of Chinese Academy of Social Sciences, Beijing, China; ^2^College of Marxism, Chongqing Medical College and Pharmaceutical College, Chongqing, China; ^3^Institute of Marxism, Central South University, Changsha, China

**Keywords:** academic misconduct, innovative behavior, academic anxiety, educational level, employment confidence, graduate student

## Abstract

**Introduction:**

Academic misconduct among graduate students poses a significant challenge in graduate education. This study aims to explore the impact of innovative behavior on graduate student academic misconduct and its underlying mechanisms.

**Methods:**

A survey was conducted on 677 graduate students currently enrolled in Beijing universities to assess their innovative behavior, academic misconduct, academic anxiety, employment confidence, educational levels, among other factors. The study analyzed the mediating roles of academic anxiety, employment confidence, and educational levels in the relationship between innovative behavior and graduate student academic misconduct.

**Results:**

Graduate student innovative behavior exerts a negative predictive effect on academic misconduct, with a stronger emphasis on innovative behavior associated with a reduced likelihood of academic misconduct. Academic anxiety plays a mediating role in the relationship between graduate student innovative behavior and academic misconduct. Educational levels and employment confidence each play moderating roles in the latter stages of the mediation effects concerning graduate student innovative behavior, academic anxiety, and academic misconduct.

**Conclusion:**

This study reveals the mediating role of academic anxiety in the relationship between innovative behavior and graduate student academic misconduct. It also identifies the moderating roles of employment confidence and educational levels. These findings deepen our understanding of the relationship between innovative behavior and graduate student academic misconduct and are conducive to preventing such misconduct among graduate students.

## 1. Introduction

Academic misconduct should be judged based on whether it violates academic norms, which refers to behaviors that contravene the principles of research integrity and breach academic standards (Muñoz-García and Aviles-Herrera, 2013; Su and Wang, 2022). Previous research has indicated that academic misconduct by graduate students can severely impact the quality of graduate education and worsen the overall research environment, hindering national technological innovation (Zhang et al., 2018). In fact, graduate student academic misconduct is a global challenge that has been on the rise alongside the expansion of higher education (Macfarlane et al., 2012). Simultaneously, due to the lack of education on academic integrity, many graduate students have a limited understanding of academic misconduct, indirectly contributing to its prevalence (Mahmud and Bretag, 2013; Sbaffi and Zhao, 2022).

In recent years, with the gradual increase in the scale of graduate admissions in China, incidents of graduate student academic misconduct have garnered widespread societal attention. How to address the issue of graduate student academic misconduct has become an urgent question that needs to be answered. In September 2020, the State Council Academic Degrees Committee and the Ministry of Education of the People’s Republic of China issued “Several Opinions on Further Strictly Regulating Degree and Graduate Education Quality Management,” which stated, “Incorporate the prevention and handling of academic misconduct into the scope of national education supervision, normalize academic integrity management, and enhance the ability to handle and respond to academic misconduct incidents promptly” (Ministry of Education of the People’s Republic of China, 2020). This reflects the government’s commitment to graduate education and addressing the issue of graduate student academic misconduct.

In response to the government’s call to address the problem of graduate student academic misconduct and to address societal concerns about graduate education, this study focuses on the issue of graduate student academic misconduct. It aims to explore the deep-seated relationship between innovative behavior and graduate student academic misconduct, with the hope of making valuable contributions to research on graduate education.

### 1.1. Relationship between innovative behavior and graduate student academic misconduct

Innovative behavior refers to the generation, introduction, or application of novel and beneficial proactive actions at any organizational level. Innovative behavior contributes to the improvement of organizational or individual efficiency. It represents the external manifestation of innovation capability, and whether innovative behavior stands out or not can effectively reflect the strength of innovation capability and creative thinking (West and Farr, 1989; Scott and Bruce, 1994; Kleysen and Street, 2001). According to self-determination theory, individuals’ behavior arises from both internal and external motivations. Internal motivation is based on self-interest and competence needs, while external motivation arises from external rewards or punishments (Ryan and Deci, 2000). Most graduate students have a strong interest in scientific research and are tasked with achieving research innovation. When graduate students exhibit prominent innovative behavior, their autonomous and competence needs are satisfied, and they can successfully complete their studies or obtain research rewards. In this context, both internal and external motivations for academic misconduct are reduced.

However, when graduate students engage in fewer innovative behaviors and cannot fulfill their academic or research goals, some may resort to academic misconduct driven by internal and external motivations (Krou et al., 2021; Yu and Zhang, 2021). Previous research has found that creative thinking has a positive impact on academic integrity, and education in creative thinking can reduce the occurrence of academic misconduct (Eshet and Margaliot, 2022). There are also studies that theoretically suggest that insufficient innovative capability among graduate students, resulting in an inability to produce valuable research outcomes, directly leads to academic misconduct (Su and Wang, 2022). Therefore, fostering academic innovation capability and practical innovation capability can help reduce the occurrence of graduate student academic misconduct (Fu, 2022). Through the above analysis, it can be seen that previous research has addressed the relationship between innovative behavior and graduate student academic misconduct but has primarily focused on theoretical analysis, lacking empirical analysis support. Based on this, this study proposes hypothesis 1.

*H1*: Innovative behavior is expected to negatively predict graduate student academic misconduct.

### 1.2. The mediating role of academic anxiety

Anxiety is a negative emotional state typically characterized by worries, tension, and unease about potential challenges or threats (Barlow, 2000). Academic anxiety, in particular, pertains to anxiety stemming from research work and shares similarities with statistical anxiety and writing anxiety. Statistical anxiety arises from the statistical knowledge and skills required in scientific research (Eshet et al., 2022), while writing anxiety is associated with paper writing in scientific research (Huerta et al., 2016). However, academic anxiety differs in that it is linked to the entirety of research work (Wang et al., 2014). Regarding the relationship between innovative behavior and academic anxiety, some scholars have found through meta-analysis that innovative behavior is significantly negatively correlated with anxiety (Baas et al., 2008), while others have discovered a negative relationship between research creativity and academic anxiety (Li et al., 2019). In terms of the relationship between academic anxiety and academic misconduct, some researchers have found that research assessment pressure is a significant influencing factor in academic misconduct in higher education institutions (Chang and Jiang, 2008). Additionally, through a literature analysis, some scholars have concluded that academic anxiety emotions can trigger academic misconduct (Ali and Aboelmaged, 2021). Furthermore, research has shown that insufficient research innovative behavior can lead to negative emotions such as stress and anxiety, which, in turn, can positively influence graduate students’ attitudes toward academic misconduct (Sun et al., 2016). Graduate students who lack innovative behavior may face the dilemma of being unable to complete their academic tasks, leading to academic anxiety, which then becomes a motivation for engaging in academic misconduct. Based on this, we propose hypothesis 2.

*H2*: Academic anxiety is hypothesized to mediate the relationship between innovative behavior and graduate student academic misconduct.

### 1.3. The moderating role of educational level

Educational level primarily refers to the level of education that individuals have received or are about to receive. For graduate student populations, this mainly includes master’s and doctoral students. Regarding the relationship between educational level and anxiety, some researchers have used longitudinal survey data analysis to find that emotions, including anxiety, accumulate throughout an individual’s life, and lower levels of educational attainment are significantly correlated with anxiety, while higher levels of educational attainment have an inhibitory effect on anxiety (Bjelland et al., 2008). There is also research indicating that younger, lower-grade students may experience greater anxiety when facing unexpected situations due to a lack of coping skills (Pelucio et al., 2022). Regarding the relationship between educational level and academic misconduct, some researchers have found that as educational levels increase, instances of academic misconduct gradually decrease, with doctoral students having almost no cases of academic misconduct (Zhou and Qin, 2014). Other studies analyzing news reports related to academic misconduct have found significantly more reports of academic misconduct among master’s students compared to doctoral students, likely due to differences in the population size between the two groups (Pan and Liu, 2019). Generally, master’s and doctoral students differ in factors such as age, program duration, the research tasks they undertake, and the difficulty of graduation, which may moderate the impact of Academic anxiety on academic misconduct. Based on this, we propose hypothesis 3.

*H3*: Educational level is expected to moderate the latter portion of the relationships among innovative behavior, research anxiety, and graduate student academic misconduct.

### 1.4. The moderating role of employment confidence

Employment confidence refers to psychological expectations regarding future employment prospects. Concerning the relationship between employment confidence and anxiety, some scholars argue that the COVID-19 pandemic has influenced the employment situation of university students and have analyzed the relationship between COVID-19 anxiety and employment confidence. They found a negative correlation between pandemic-related anxiety and university students’ employment confidence (Zheng et al., 2022). Other research has indicated that employment uncertainty can lead to increased individual anxiety, while having confidence in job stability helps reduce anxiety (Chan et al., 2021). Regarding the relationship between employment confidence and academic misconduct, some researchers have analyzed that in situations with unclear job prospects, some graduate students, lacking confidence in their employability, may resort to academic misconduct to enhance their competitiveness in the job market (Li and Zhao, 2019). Some scholars further point out that while academic misconduct can impact students’ employability when exposed, it is often difficult to detect in the short term. Therefore, driven by the external motivation to secure good employment, some graduate students may choose academic misconduct (Luck et al., 2022). Analysis reveals a strong correlation between employment confidence, academic anxiety, and graduate student academic misconduct, suggesting that employment confidence may play a moderating role in the relationship between academic anxiety and graduate student academic misconduct. Based on this, we propose hypothesis 4.

*H4*: Employment confidence is anticipated to moderate the latter portion of the relationships among innovative behavior, academic anxiety, and graduate student academic misconduct.

## 2. Research model

**Figure 1:**
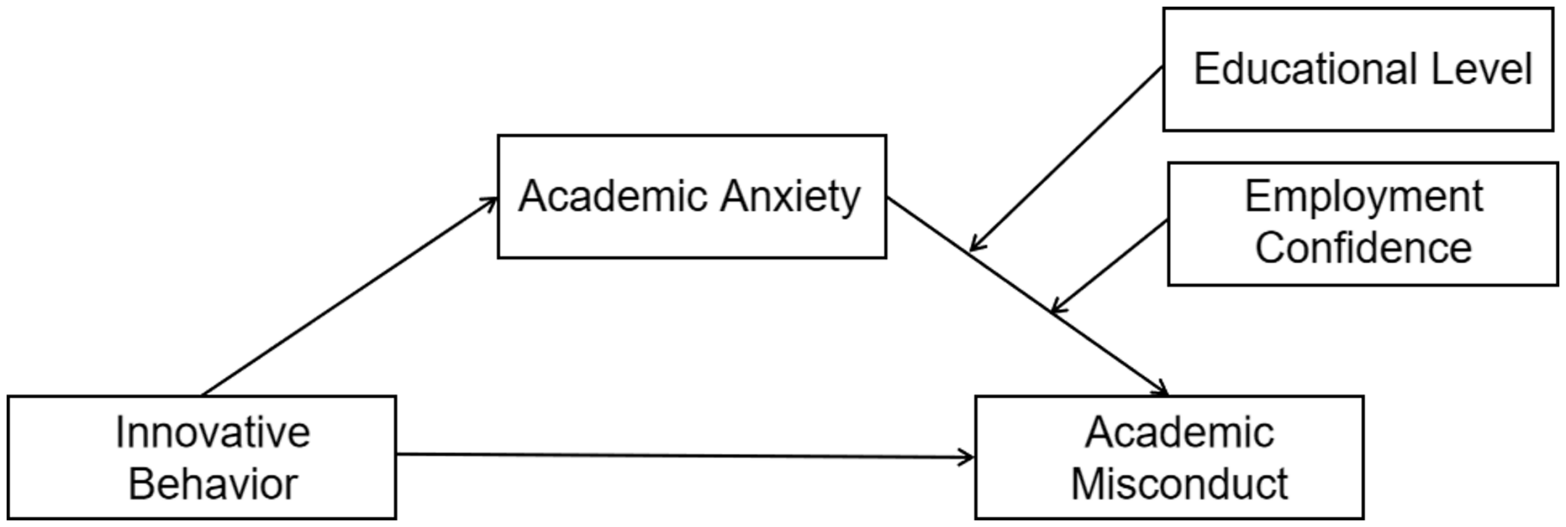
Moderated mediation model.

In summary, existing research has focused on the relationship between innovative behavior and academic misconduct but has primarily concentrated on theoretical analysis, with limited empirical research outcomes. Therefore, this study employs self-determination theory, with a primary focus on investigating the impact of innovative behavior on graduate student academic misconduct. Additionally, it explores the mediating role of academic anxiety and analyzes the moderating effects of educational level and employment confidence in the relationship between academic anxiety and graduate student academic misconduct (see Figure 1).

## 3. Research design

### 3.1. Object of study

Convenient sampling was employed in this study. From January to February 2023, paper and electronic questionnaires were distributed to graduate students in three universities in Beijing, and 744 questionnaires were collected, of which 677 were valid. In terms of gender, female postgraduates account for 51.8%, and male postgraduates account for 48.2%; in terms of household registration, rural postgraduates accounted for 55.4%, and non-rural postgraduates accounted for 44.6%;In terms of educational level, 67.7% of them are postgraduates with a master’s degree, and 32.3% are postgraduates with a doctoral degree; in terms of specialty, 63.5% are postgraduates majoring in science, engineering, agriculture and medicine, and 36.5% in humanities and social sciences.

### 3.2. Research tools

The research tools mainly include the explanatory variables of academic misconduct scale, the explanatory variables of innovative behavior scale, academic anxiety scale, employment confidence questionnaire and so on, and the reliability and validity of each scale are good.

#### 3.2.1. Academic misconduct scale

It is generally difficult to obtain accurate data by directly measuring academic misconduct, so this study uses the research misconduct scale compiled by Zhao et al. (2012), which can measure the possibility of academic misconduct. The scale consists of two dimensions of science-oriented and relationship-oriented research misconduct, a total of 10 items, and uses Likert five-point scoring. The higher the score, the greater the possibility of academic misconduct. In this study, the Cronbach’s alpha coefficient of the scale was 0.835, this suggests that the scale has good internal consistency.

#### 3.2.2. Innovative behavior scale

Innovative behavior refers to the behavior full of creativity and novelty. To measure graduate student innovative behavior, we adapted the Innovative Behavior Scale developed by Scott and Bruce (1994). It has been used by domestic scholars in the study of innovative behavior of graduate students with good reliability and validity (Zhang et al., 2022). There are six items in the scale, and the higher the score, the more significant the innovative behavior. In this study, the Cronbach’s alpha coefficient of the scale was 0.800, this suggests that the scale has good internal consistency.

#### 3.2.3. Academic anxiety scale

Academic anxiety refers to the anxiety and anxiety caused by scientific research work. The academic anxiety scale designed by Wang et al. (2014). Was used, which consisted of four items and was scored by Likert five points. The higher the score, the stronger the academic anxiety. In this study, the Cronbach’s alpha coefficient of the scale was 0.724, this suggests that the scale has good internal consistency.

#### 3.2.4. Employment confidence questionnaire

In assessing employment confidence, this study referred to previous employment confidence questionnaires (Zheng et al., 2022). To examine the employment confidence of graduate students with the self-compiled question “When graduate students graduate, do you have the confidence to find a desired job?,” set the score of Likert Grade 7, 1 represents very little confidence, 7 represents very confident, the higher the score, the stronger the employment confidence.

### 3.3. Statistical methods

Data statistical and analytical procedures were conducted using SPSS 26 software and the Model 4 and Model 14 from the PROCESS macro plugin, from a professional academic perspective.

## 4. Results

### 4.1. Deviation analysis of homologous common methods

The data of this study are all from the self-assessment of graduate students, and there may be a common method bias problem, so the common method bias test should be carried out on the variable data. Harman single factor test method was used, and the test results showed that the variance percentage of the first principal component was 36. 61%, which reached the qualified standard of less than 40% (Podsakoff and Organ, 1986). Therefore, it can be judged that there is no outstanding common method bias problem in this study.

### 4.2. Descriptive statistics and correlation analysis

The mean value, standard deviation and correlation analysis were performed for each variable, and the analysis results are shown in Table 1. The results show that the average score of the academic misconduct scale is 18.41, and combined with the range of values (10–50), it can be judged that the overall integrity of graduate research is good, and the possibility of academic misconduct is low;The average score of the Graduate Student Innovative Behavior Scale is 23.77, combined with the range of values (6–30), it can be judged that most graduate students have good innovative behavior. From the correlation analysis, innovative behavior, academic anxiety and academic misconduct among graduate students are significantly correlated, but the specific impact path remains to be further analyzed and tested.

**Table 1:** Results of descriptive statistics and correlation analysis.

	Mean	SD	1	2	3
1. Innovative behavior	23.77	4.32	–		
2. Academic anxiety	7.60	2.01	−0.32***	–	
3. Academic misconduct	18.41	5.57	−0.68***	0.52***	–

*p < 0.05, **p < 0.01, ***p < 0.001.

### 4.3. Hypothesis testing

Hierarchical regression analysis and Bootstrap method were used for hypothesis testing, and the PROCESS plug-in written by Hayes (2015) was used to analyze and process the data in SPSS.

Main effect test. The results of M3 analysis (see Table 2) show that the innovative behavior of graduate students can significantly and negatively affect the academic misconduct among graduate students (*p* < 0.001). The more prominent the innovative behavior of graduate students is, the lower the possibility of academic misconduct is. Hypothesis 1 passes the test.

**Table 2:** Hierarchical regression analysis results.

	Academic anxiety	Academic misconduct						
	M1	M2	M3	M4	M5	M6	M7	M8
Gender	−0.157	−0.514	−0.480	−0.331	−0.368	−0.294	−0.486	−0.427
Household registration	0.182	0.658	0.353	0.180	0.301	0.305	0.364	0.394
Major	0.278	−0.447	−0.062	−0.326	−0.324	−0.414	−0.295	−0.327
Innovative behavior	−0.150***		−0.871***	−0.729***	−0.730***	−0.733***	−0.722***	−0.724***
Academic anxiety				0.949***	0.938***	1.683***	0.951***	1.597***
Educational level					−0.918**	3.238**		
Employment confidence							−0.491***	0.695*
Academic level × academic anxiety						−0.551***		
Employment confidence × academic anxiety								−0.159***
*R*^2^	0.109	0.008	0.462	0.567	0.573	0.582	0.580	0.588
*F*	20.611***	1.759	144.522***	175.737***	149.741***	132.957***	154.232***	136.171***

*p < 0.05, **p < 0.01, ***p < 0.001; unstandardized coefficients *β* was used for hierarchical regression analysis.

Test of mediating effect. The results of M1 analysis showed that the innovative behavior of graduate students had a significant negative impact on academic anxiety (*p* < 0.001). M4 analysis showed that academic anxiety had a significant positive effect on academic misconduct (*p* < 0.001);Compared with M3, academic anxiety was added to M4, and the regression coefficient of graduate students’ innovative behavior on graduate students’ academic misconduct changed, but it was still significant (*p* < 0.001). At the same time, standardization was applied to the independent variables, dependent variables, and mediator variables. The Bootstrap test showed that the mediation effect was significant, and the mediation effect value was −0.110, 95% CI = [−0.145, −0.078], excluding 0, as shown in Table 3.It can be seen that academic anxiety plays a partial mediating role between graduate students’ innovative behavior and academic misconduct. Hypothesis 2 passes the test.

**Table 3:** Total effect, direct effect and mediating effect (Bootstrap = 5,000).

	Effect value	Standard error	LL 95% CI	UL 95% CI	Relative effect value
Total effect	−0.676	0.028	−0.731	−0.620	
Direct effect	−0.566	0.027	−0.619	−0.513	83.728%
Mediating effect	−0.110	0.017	−0.145	−0.078	16.272%

Moderating effect test. The results of M6 analysis showed that the interaction of academic level and academic anxiety had a significant negative impact on academic misconduct of postgraduates (*p* < 0.001). Previous studies have shown that the first type of error rate tested in turn is low, and if the test result is significant, it can be known that the mediation effect is moderated (Wen and Ye, 2014), so it can be judged that the educational level plays a moderating role in the mediation process of innovative behavior, academic anxiety and academic misconduct of postgraduates. Assumption 3 passes the test.

**Figure 2:**
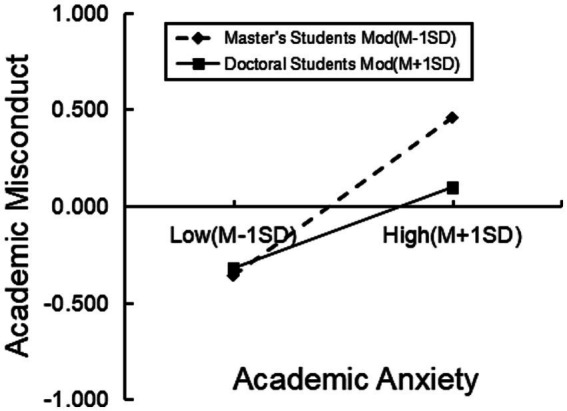
Moderating effect of educational level.

Furthermore, to explore the moderating effect of educational level, the educational level was divided into two groups - master’s students and doctoral students - based on one standard deviation above and below the mean. Simple slope analysis was conducted to delve deeper into this. Through the simple slope analysis results (see Figure 2), it was observed that both the master’s student group (simple slope = 0.41, *p* < 0.001) and the doctoral student group (simple slope = 0.21, *p* < 0.001) enhanced the influence of academic anxiety on academic misconduct. However, the reinforcing effect was smaller in the doctoral student group, indicating that a higher educational level can to some extent mitigate the impact of academic anxiety on academic misconduct.

The results of M8 analysis showed that the interaction of employment confidence and academic anxiety had a significant negative impact on academic misconduct of postgraduates (*p* < 0.001). Therefore, it can be judged that employment confidence plays a moderating role in the mediation process of innovative behavior, academic anxiety and academic misconduct among graduate students. Assumption 4 passes the test.

**Figure 3:**
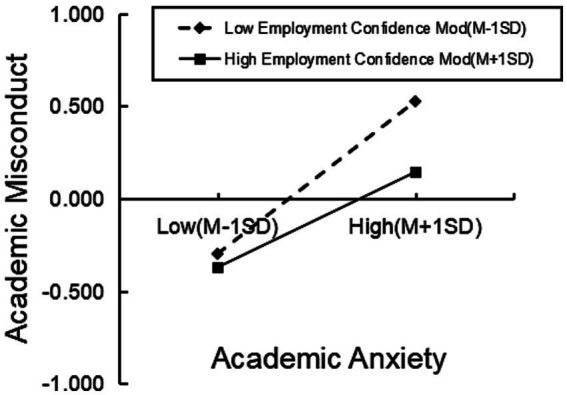
Moderating effect of employment confidence.

To further investigate the moderating effect of employment confidence, educational levels were divided into low employment confidence and high employment confidence groups based on one standard deviation above and below the mean. Simple slope analysis was conducted for this purpose. Through the results of the simple slope analysis (see Figure 3), it was found that both the low employment confidence group (simple slope = 0.41, *p* < 0.001) and the high employment confidence group (simple slope = 0.26, *p* < 0.001) intensified the impact of academic anxiety on academic misconduct. However, compared to graduate students with low employment confidence, those with high employment confidence exhibited a weaker influence of academic anxiety on academic misconduct, indicating that high employment confidence can mitigate the impact of academic anxiety on academic misconduct.

## 5. Discussion

This study examined the impact of innovative behavior on graduate student academic misconduct, explored the mediating role of academic anxiety, and investigated the moderating effects of educational level and employment confidence.

The research findings indicate that innovative behavior can negatively predict graduate student academic misconduct, hypothesis 1 passes the test. In other words, the more prominent a graduate student’s innovative behavior, the lower the likelihood of academic misconduct, while less notable innovative behavior is associated with a higher likelihood of academic misconduct. This aligns with previous research findings (Maloshonok and Shmeleva, 2019; Diotaiuti et al., 2021). Self-determination theory provides a robust theoretical framework for understanding these results. It posits that individual behavior is driven by internal motivations such as autonomy and competence needs, as well as external motivations such as rewards and punishments. When both internal and external motivations are sufficiently met, they influence an individual’s behavior (Gagné and Deci, 2005). Graduate students engage in research work, fundamentally an innovative endeavor. Prominent innovative behavior often leads to more research output, satisfying autonomy and competence needs, which in turn reduces the internal motivation for academic misconduct. Furthermore, higher research output often implies that graduate students can successfully complete their studies or obtain research rewards, diminishing the external motivation for academic misconduct. However, when graduate students exhibit insufficient innovative behavior, their autonomy and competence needs remain unmet, and they may struggle to complete their studies (Krou et al., 2021; Yu and Zhang, 2021), thus fostering a stronger internal and external motivation for academic misconduct (Su and Wang, 2022). Given that innovative behavior is an external manifestation of innovative capabilities, institutions involved in graduate education, such as universities, should actively encourage research training to enhance the innovative capabilities of graduate students. As their innovative capabilities grow and innovative behavior becomes more pronounced, graduate students are less inclined to engage in academic misconduct (Fu, 2022).

The study also found that academic anxiety mediates the relationship between innovative behavior and graduate student academic misconduct, hypothesis 2 passes the test. In other words, innovative behavior can directly influence academic misconduct and indirectly affect it by impacting academic anxiety. This finding is consistent with previous research (Sun et al., 2016; Ali and Aboelmaged, 2021). When graduate students exhibit inadequate innovative behavior and consequently produce fewer research outcomes, this may affect their award and recognition prospects. Driven by external motivations, academic anxiety tends to emerge. Simultaneously, insufficient innovative behavior reflects an inability to effectively fulfill research responsibilities. When peers are better equipped to handle research tasks, it can exacerbate anxiety among individuals (Pisarik et al., 2017). If adverse emotions like anxiety are not effectively managed, they can further impact academic performance (Diotaiuti et al., 2021). Consequently, graduate institutions should prioritize the psychological well-being of their students, offering diverse psychological counseling services and encouraging peer support among graduate students to reduce academic anxiety and, by extension, decrease the occurrence of academic misconduct.

The study found that educational level can moderate the latter part of the pathway involving innovative behavior, academic anxiety, and the mediating effect on graduate student academic misconduct, hypothesis 3 passes the test. In comparison to lower educational levels, higher educational levels suppress the influence of academic anxiety on graduate student academic misconduct. This finding aligns with previous research (Bjelland et al., 2008; Zhou and Qin, 2014). Possible reasons for this include the likelihood that higher educational level doctoral students have a better understanding of academic norms and a clearer awareness of the harmful consequences of academic misconduct. Thus, even when experiencing high academic anxiety, they are less likely to resort to academic misconduct. In contrast, master’s students at lower educational levels may have a limited understanding of academic norms and may not recognize certain behaviors that violate these norms as academic misconduct (Burgason et al., 2019). Consequently, when experiencing high academic anxiety, they are more likely to engage in academic misconduct (Su and Wang, 2022). Additionally, lower educational level master’s students, who are often engaging in academic or research work for the first time, may lack the necessary coping skills to handle innovative research tasks, making them more susceptible to anxiety (Pelucio et al., 2022). In contrast, doctoral students typically have more training, including at the master’s level, and thus possess stronger coping skills, reducing the likelihood of experiencing anxiety. Therefore, preventing academic misconduct should focus on the graduate student population with lower educational levels. These students are relatively new to academia, may have unclear perceptions of academic norms, and insufficient understanding of the consequences of academic misconduct. Institutions involved in graduate education should emphasize the importance of adhering to academic norms and understanding the dangers of academic misconduct throughout the training process, especially for students with lower educational levels. This can be achieved through increased awareness and guidance.

The study also discovered that employment confidence can moderate the latter part of the pathway involving innovative behavior, academic anxiety, and the mediating effect on graduate student academic misconduct, hypothesis 4 passes the test. In comparison to lower employment confidence, graduate students with higher employment confidence suppress the influence of academic anxiety on graduate student academic misconduct. This finding aligns with previous research (Li and Zhao, 2019). The potential reason behind this is that graduate students with higher employment confidence have a positive outlook on their future job prospects and believe they can secure desirable employment. As a result, they are motivated to avoid academic misconduct to prevent potential negative impacts on their future careers. Consequently, they lack the motivation for academic misconduct, effectively inhibiting the impact of academic anxiety. On the other hand, graduate students with lower employment confidence may believe they will not secure their desired jobs. In situations of high academic anxiety, the likelihood of academic misconduct increases as they seek to improve their employment prospects (Luck et al., 2022). Furthermore, while academic pressure or the need for higher academic performance can lead to academic misconduct (Bayaa Martin Saana et al., 2016), higher employment confidence can reduce negative emotions like anxiety (White et al., 2018). Therefore, when employment confidence is higher, the negative emotions resulting from academic pressure or the need for high academic performance are reduced, subsequently decreasing the occurrence of graduate student academic misconduct. Consequently, graduate institutions should enhance career guidance for graduate students, especially in contexts of employment uncertainty. They should provide support, guidance, and assistance to help graduate students plan their careers effectively, ultimately boosting their employment confidence (Chan et al., 2021).

## 6. Limitations and future prospects

While this study has yielded some exploratory findings, it still has certain limitations. Firstly, the sample size was relatively small. Due to constraints in research resources and time, the sample size in this study was limited, which could potentially impact the generalizability of the research results. Therefore, future research endeavors may consider expanding the sample size to enhance its representativeness. Secondly, this study utilized cross-sectional data and did not conduct longitudinal tracking surveys, leaving causal relationship models in need of further examination. Consequently, in future research, longitudinal surveys could be conducted to analyze the causal relationships between variables longitudinally. This would improve the scientific rigor and persuasiveness of the research.

## Data availability statement

The raw data supporting the conclusions of this article will be made available by the authors, without undue reservation.

## Ethics statement

The studies involving humans were approved by Ethics Review Committee of Renmin University of China. The studies were conducted in accordance with the local legislation and institutional requirements. The participants provided their written informed consent to participate in this study. Written informed consent was obtained from the individual(s) for the publication of any potentially identifiable images or data included in this article.

## Author contributions

PS: Conceptualization, Data curation, Methodology, Writing - original draft, Investigation. MH: Supervision, Writing – review & editing, Funding acquisition.
